# Severe bronchial obstruction and lung atelectasis following suspected massive inhalation of gluten-free flour: a case report

**DOI:** 10.3389/fped.2026.1983146

**Published:** 2026-10-26

**Authors:** Federica Porcaro, Antonella Coretti, Roberto Grandinetti, Maria Giovanna Paglietti, Alessandra Ottavianelli, Giovanni Cristalli, Renato Cutrera

**Affiliations:** 1 Pediatric Pulmonology & Cystic Fibrosis Unit, Respiratory Research Unit, Bambino Gesù Children’s Hospital, IRCCS, Rome, Italy; 2 Diagnostic and Interventional Radiology, Bambino Gesù Children’s Hospital, IRCCS, Rome, Italy; 3 Unit of Otorhinolaryngology, Bambino Gesù Children’s Hospital, IRCCS, Rome, Italy

**Keywords:** acute respiratory failure, bronchoscopy, case report, complete lung atelectasis, gluten-free flour, inhalation, pediatric age

## Abstract

Acute respiratory failure in children is most commonly caused by respiratory infections, asthma, foreign body aspiration, trauma, and disorders affecting the respiratory pump or the central control of breathing. We describe an unusual and underreported cause of severe respiratory compromise in a previously healthy 2-year-old boy. The patient presented with acute hypoxemic respiratory failure that was poorly responsive to inhaled bronchodilators, systemic corticosteroids, and oxygen therapy, requiring endotracheal intubation and invasive mechanical ventilation. Extensive diagnostic investigations did not identify a common infectious cause or a conventional aspirated foreign body. Chest imaging demonstrated complete left lung atelectasis and right upper lobe atelectasis. Flexible bronchoscopy with therapeutic bronchoalveolar lavage revealed and removed abundant white, highly viscous endobronchial material, resulting in progressive re-expansion of the affected lung and marked clinical improvement. The child was successfully extubated and discharged from the hospital after 20 days of hospitalization. A subsequent review of the clinical history identified the likely precipitating event: inhalation of gluten-free flour after accidental activation of a planetary mixer during cake preparation. We hypothesize that inhalation of a large amount of powder contributed to airway obstruction through direct particulate deposition and/or secondary inflammatory mucus formation; hydration of a hydrocolloid component may also have contributed to the marked viscosity of the endobronchial material, although this mechanism could not be directly demonstrated. This case highlights the importance of meticulous history-taking in identifying rare causes of acute respiratory failure and illustrates how seemingly innocuous environmental exposures may result in life-threatening airway obstruction requiring prompt recognition and intervention.

## 1 Introduction

The most common causes of acute respiratory failure in children include respiratory infections, asthma, trauma, and disorders affecting either the respiratory pump or the central control of breathing, interstitial lung diseases or anaphylaxis (1, 2).

Foreign body (FB) aspiration into the respiratory tract is another cause of acute respiratory failure and occurs predominantly in children aged 1–3 years (3). Respiratory FBs are more commonly of organic than inorganic origin (4). Several studies have reported that the right bronchial tree is the most common site of FB impaction (3).

Complications of FB aspiration include pneumonia, pulmonary atelectasis, lung consolidation, lung abscess, pneumothorax, and bronchiectasis. Life-threatening complications and adverse outcomes have also been reported (3). Although imaging findings may be inconclusive, bronchoscopy remains the diagnostic and therapeutic procedure of choice when there is a high clinical suspicion of FB aspiration (5).

Most FBs are removed from the respiratory tract within the first 24 h, and prompt management is essential to reduce the risk of the aforementioned complications.

Although a history of witnessed aspiration provided by the parents is often helpful in establishing the diagnosis, FB aspiration in the absence of a suggestive history remains a significant clinical challenge. This is particularly relevant when aspiration occurs in seemingly harmless circumstances that may nevertheless result in severe respiratory compromise requiring emergency intervention. Accidental inhalation of fine powders represents a much less frequently reported cause of severe respiratory compromise in children. The available pediatric literature consists predominantly of case reports and small case series involving substances such as talcum powder, starches, and other particulate materials, and reliable incidence data are therefore unavailable. Clinical manifestations may result from direct particulate obstruction, inflammatory airway injury, or a combination of these mechanisms. Unlike conventional foreign body aspiration, no specific management guidelines are available for accidental food-powder inhalation, and treatment is generally individualized according to the severity of respiratory compromise and bronchoscopic findings.

We report the case of a previously healthy 2-year-old boy who developed severe hypoxemic respiratory failure and extensive bronchial obstruction following suspected massive inhalation of a gluten-free flour mixture.

Written permission was obtained from the patient’s parents to publish this case and the corresponding imaging studies, and the Scientific Directorate of Bambino Gesù Children’s Hospital approved the manuscript content and its submission.

## Case description

We report the case of a 2-year-old boy, admitted to our hospital with sudden and acute respiratory failure with rapid deterioration of clinical conditions requiring intensive care. He was pale, irritable, tachypneic (respiratory rate 62 breaths/min) and dyspneic with oxygen saturation of 55% on room air. Chest auscultation revealed absent breath sounds over the left hemithorax. Examination of the oral cavity showed no evidence of foreign bodies, and both parents denied any history of trauma or events suggestive of foreign body aspiration during play or meals in the preceding hours. Arterial blood gas analysis was consistent with acute hypoxemic respiratory failure (type I) associated with mild metabolic acidosis, likely due to tissue hypoxia (pH 7.34, pCO₂ 39 mmHg, pO₂ 39 mmHg, HCO₃⁻ 20.5 mmol/L, lactate 3.5 mmol/L). Laboratory investigations revealed leukocytosis with neutrophilia (white blood cell count 20.08 × 10^3^/μL; neutrophils 13.75 × 10^3^/μL) and mildly elevated C-reactive protein (0.79 mg/dL).

Patient's family history was notable for atopy and celiac disease in his mother, whereas his past medical history was unremarkable. The postnatal period was uneventful, and he had experienced neither bronchiolitis during the first year of life nor recurrent respiratory infections. The child did not attend daycare or school and had a regular height and weight growth.

On an afternoon in January 2026, his mother noticed the progressive onset of respiratory distress, which was not preceded by symptoms of an upper respiratory tract infection and did not respond to home treatment with inhaled salbutamol, inhaled corticosteroids, and systemic betamethasone initially prescribed by general pediatrician based on the assessment carried out at home. His condition rapidly progressed to acute respiratory failure, and he was immediately brought to the Emergency Department (ED) of our hospital.

A figure showcasing a timeline with relevant data from the episode of care is available (Figure 1).

**Figure 1:**
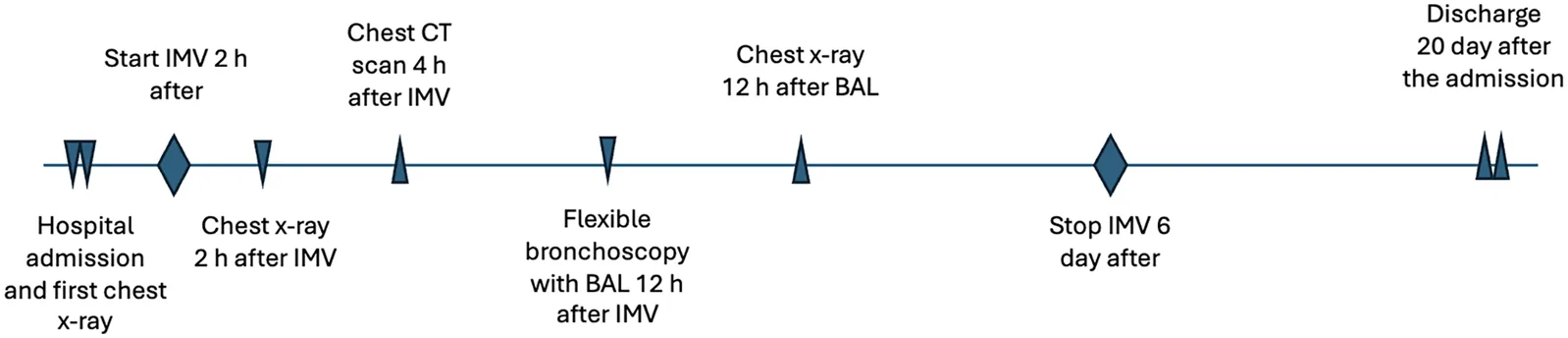
Timeline of relevant data from the episode of care.

Upon arrival, the patient appeared critically ill. He was immediately treated with oxygen delivered at 10 L/min via bag-valve-mask ventilation. Intravenous methylprednisolone (2 mg/kg) was administered; however, because of the lack of clinical improvement and progressive respiratory instability, endotracheal intubation became necessary in the ED.

Chest radiography demonstrated right upper lobe atelectasis and complete opacification of the left lung. A small radiopaque opacity in the left upper parahilar region together with a linear hyperlucent area in the left lower paramediastinal region raised the suspicion of pneumothorax (Figure 2).

**Figure 2:**
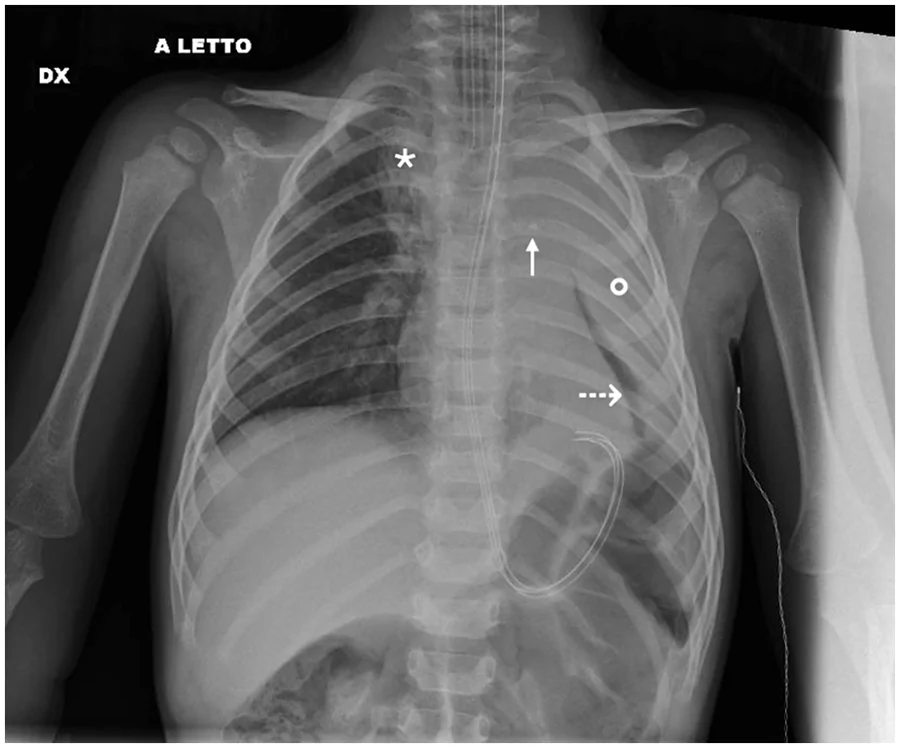
First chest x-ray showing: 1) right upper lobe atelectasis (asterisk); 2) complete opacification of the left lung (spot), 3) a small radiopaque opacity in the left upper parahilar region (arrow), and 4) a linear hyperlucent area in the left lower paramediastinal region suspected for pneumothorax (dotted arrow).

Mechanical ventilation was started in addition to broad-spectrum intravenous antibiotics (ceftriaxone), inhaled salbutamol and corticosteroids, and systemic methylprednisolone. A follow-up chest radiograph obtained within the following 2 h showed persistent complete opacification of the left lung.

Chest computed tomography (CT) was performed 4 h after initiation of IMV to better characterize the pulmonary findings, assess the extent of the suspected pneumothorax, and rule out an aspirated foreign body.

CT imaging demonstrated a patent trachea and right main bronchus, whereas the left main bronchus was abruptly interrupted shortly after its origin by apparent endoluminal material; complete left lung atelectasis and right upper lobe atelectasis were confirmed; interstitial emphysema was observed within the ventilated portions of the right lung, together with extensive pneumomediastinum predominantly involving the lower paraesophageal and left paracardiac/retrocardiac regions; both pulmonary hila appeared globular and enlarged, raising the possibility of hilar lymphadenopathy. This finding was not evident on chest radiographs obtained before or after the acute event; because no follow-up CT was clinically indicated, its nature and clinical significance could not be further characterized. (Figure 3). The radiopaque finding observed on chest radiography was interpreted as calcification of a mildly redundant ductus arteriosus.

**Figure 3:**
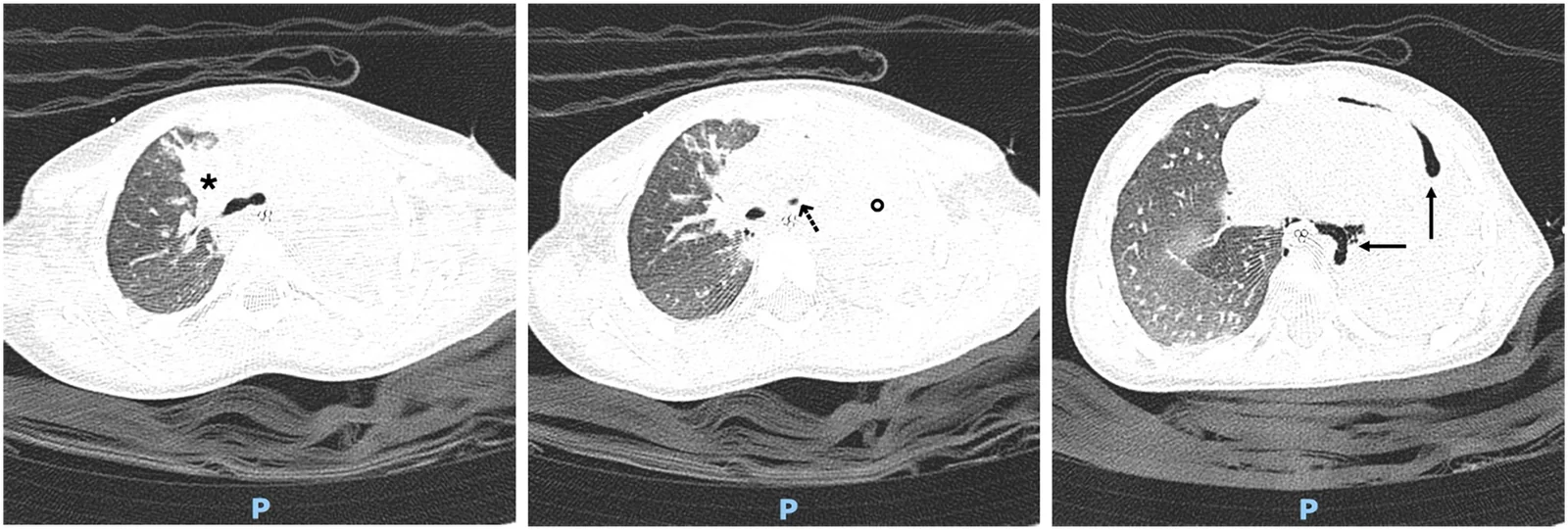
Chest CT scan showing 1) right upper lobe atelectasis (asterisk); 2) interruption of left main bronchus by apparent endoluminal material (dotted arrow) and complete left lung atelectasis (spot); 3) extensive pneumomediastinum involving the lower paraesophageal and left paracardiac/retrocardiac regions (arrows). .

In view of the imaging findings, the lack of satisfying clinical improvement, and persistent inadequate ventilation despite invasive mechanical ventilation (arterial blood gas: pH 7.29, pCO₂ 49 mmHg, pO₂ 81 mmHg, HCO₃⁻ 22 mmol/L, lactate 1.2 mmol/L), flexible bronchoscopy was performed 12 h after initiation of invasive ventilatory support, because the patient had presented during the night, and the initial priority was stabilization of his critical respiratory condition. Bronchoscopy was subsequently organized in coordination with the on-call otolaryngology team once adequate clinical stabilization had been achieved with two objectives: (1) to identify the nature of the endobronchial obstruction, and (2) to perform therapeutic bronchoalveolar lavage (BAL).

Bronchoscopy excluded the presence of a foreign body and other mass occupying space, but revealed abundant white, thick, highly viscous, and elastic mucus involving both bronchial hemisystems, with complete obstruction of the left main bronchus. The material did not reproduce the branching morphology or anatomy of the bronchial tree. It was successfully removed by bronchoscopic aspiration and lavage, and samples were sent for microbiological and cytological analyses.

All microbiological investigations, including bacterial, viral, fungal, and mycobacterial cultures, yielded negative results. Cytological examination demonstrated fibrin and inflammatory exudate, with neutrophil and eosinophil granulocytes and foamy macrophages consistent with an acute inflammatory process.

Similarly, virological and bacteriological investigations performed on upper respiratory tract specimens, including the film-array pneumonia panel, multiplex real-time PCR assays for *Mycoplasma pneumoniae* and *Chlamydia pneumoniae*, and the interferon-gamma release assay (IGRA) for tuberculosis, were all negative.

Subsequent chest radiographs showed progressive re-expansion of the left lung (Figure 4). The patient's respiratory function gradually improved, allowing successful extubation 6 days after admission in the Intensive Care Unit (ICU). Arterial blood gas analysis at that time showed pH 7.48, pCO₂ 31 mmHg, pO₂ 96 mmHg, HCO₃⁻ 24 mmol/L, and lactate 1.1 mmol/L.

**Figure 4:**
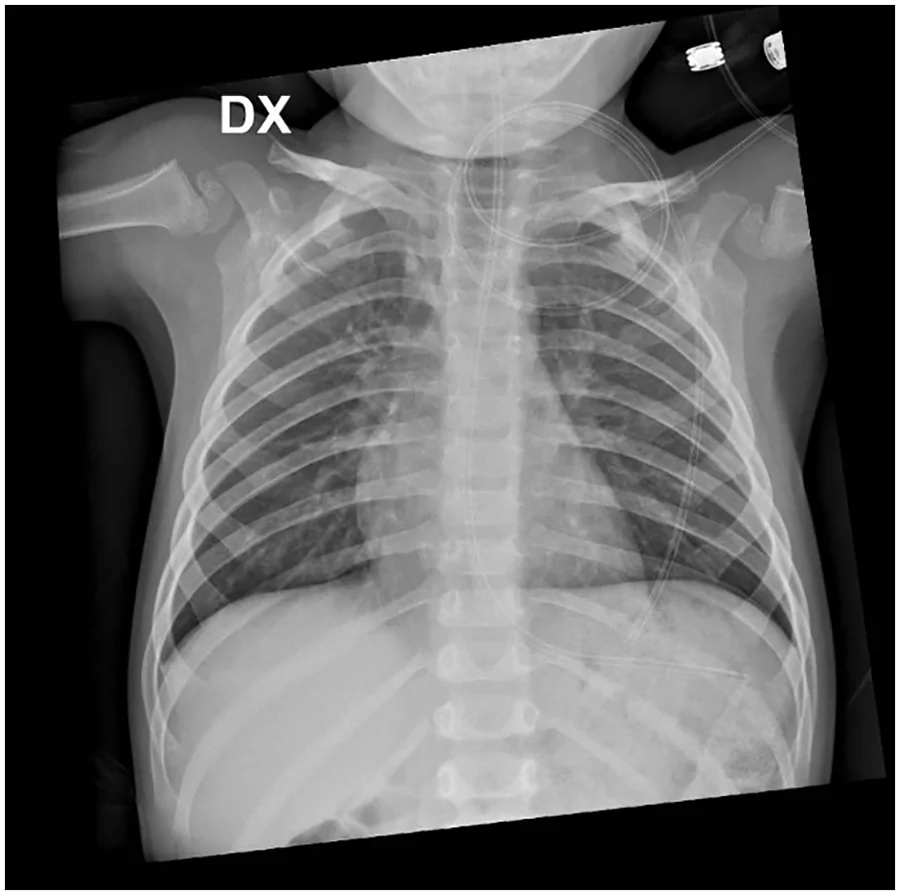
Chest x-ray performed 17 days after airway endoscopy showing complete resolution of atelectasis of right upper lobe and left lung.

Given the clinical and radiographic improvement, repeat bronchoscopy or chest CT scan was deemed unnecessary.

Following clinical stabilization, a more detailed review of the history revealed a potentially relevant exposure approximately 12 h before the onset of progressive respiratory distress. The mother recalled that, while using a planetary mixer during cake preparation, a large amount of gluten-free flour became airborne in the kitchen. The child, who was nearby and participating in the preparation, developed sudden and persistent coughing immediately after the flour became airborne. No choking episode related to food or aspiration of a solid foreign body was witnessed.

The child was discharged from the hospital after 20 days of hospitalization in good clinical condition, with inhaled corticosteroid therapy (beclomethasone 100 μg/day) prescribed to reduce residual bronchial inflammation following the event in the following three months. At the 2-month follow-up, the patient remained asymptomatic from a respiratory standpoint and required neither inhaled salbutamol nor systemic corticosteroid therapy.

## Discussion

This case illustrates an unusual cause of severe acute airway obstruction in a previously healthy child. At initial presentation, the family denied a witnessed choking episode during meals or play and there was no history suggestive of aspiration of a conventional solid foreign body. Only after stabilization and repeated history-taking was the preceding powder exposure identified, with immediate coughing after the flour became airborne followed by progressive respiratory deterioration approximately 12 h later.

The temporal association between exposure and respiratory deterioration, the bronchoscopic demonstration of extensive obstructing material, the absence of an alternative infectious or conventional foreign-body cause, and the marked improvement following bronchoscopic airway clearance support a possible causal relationship with powder inhalation. However, the precise mechanism responsible for the obstruction cannot be established from the available findings.

The mixture used for cake preparation consisted of corn starch, corn flour, and carob seed flour, the latter being a hydrocolloid thickener that rapidly form a gel upon contact with water.

We hypothesized that inhalation of a large amount of this flour containing thickener contributed to the development of acute respiratory failure through at least three mechanisms. First, inhaled particulate material may have directly contributed to mechanical airway obstruction. Second, the powder may have triggered an acute local inflammatory response with production of highly viscous mucus or cast-like material. Third, hydration of the hydrocolloid component of the flour may have increased the viscosity and cohesiveness of the endobronchial secretions. The available evidence does not allow the relative contribution of these mechanisms to be determined.

In the absence of other identifiable causes, given the temporal relationship between the events and the detection of viscous mucous and inflammatory infiltrate on cytologic examination, this was deemed the most plausible explanation.

Cases of respiratory involvement secondary to powder inhalation are reported in pediatric age in the literature.

Historically, talcum powder, which was widely used in the past for infant skin care, has been the most frequently reported inhaled powder (6).

However, other powders considered to have a lower potential for respiratory toxicity, such as corn starch powder, have also been widely used for routine infant skin care as an alternative to talcum powder. Severe respiratory disease requiring intensive treatment has been reported following accidental inhalation of both talcum and rice starch powder (6, 7).

Most reported cases involved infants, generally younger than 2 years, with symptoms developing shortly after exposure. However, delayed symptom onset has also been described. Pfenninger and D’Apuzzo reported a case of a 7-month-old girl who developed severe respiratory distress 3–4 h after accidental talcum powder inhalation (8).

A case more closely resembling the present one was reported in a previously healthy preschool-aged boy who accidentally inhaled cake decorating powder at home and subsequently developed respiratory distress and lung injury related to zinc and copper aspiration included in the powder (9).

Secondary inflammatory bronchial cast formation represents another relevant differential diagnosis. Plastic bronchitis is characterized by the formation of cohesive endobronchial casts that may reproduce the branching architecture of the bronchial tree and may contain fibrin and inflammatory cells. In our patient, cytological examination demonstrated fibrin and inflammatory cells, which could be compatible with an inflammatory response; however, the removed material did not reproduce the bronchial anatomy or show a branching cast-like morphology. These findings make classical plastic bronchitis less likely. Nevertheless, because the specimen underwent cytological rather than dedicated histopathological assessment of bronchial cast architecture, an acute inflammatory cast-like process triggered by powder inhalation cannot be completely excluded.

With regard to the potential pulmonary effects of thickening agents, a study carried out on murine models investigated the effect of airway instillation of xanthan gum-based thickener. Repeated aspiration of small amounts of the thickened water resulted in significant pulmonary inflammation also evident 10 days after the last aspiration event, suggesting a potentially prolonged inflammatory effect of the xanthan gum-based thickener (10).

The clinical effects of powder inhalation depend on the physicochemical characteristics and composition of the powder, the amount of exposure, the degree of aspiration, and the individual host response.

There are currently no specific guidelines for the management of accidental food-powder inhalation. Management is primarily supportive and should be guided by the severity of respiratory impairment. In cases of acute respiratory compromise, appropriate resuscitative measures and oxygen supplementation should be provided, while bronchodilator therapy may be considered in the presence of bronchospasm. Further interventions should be tailored to the specific substance involved and the clinical presentation.

Respiratory manifestation resulting from IgE-mediated responses to inhaled allergens include rhinitis, occupational asthma, eosinophilic bronchitis, and, in severe cases, anaphylaxis (11–13). Although these conditions are more commonly reported in workers who have significant repetitive exposure to allergen, Alonso et al. reported a case of a 2-year-old boy who suffered from asthma, urticaria and atopic dermatitis for twelve months, and developed asthma and rhinitis after manipulating wheat flour for 10 min (14).

In our patient, an IgE-mediated allergic mechanism was considered unlikely given the absence of a personal history suggestive of atopy, the previous uneventful exposure to the same gluten-free flour on other occasions, and the temporal relationship between exposure and symptom onset. Overall, the clinical presentation was considered more consistent with a direct irritant or inflammatory response to powder inhalation rather than an IgE-mediated allergic reaction.

## Conclusions

To the best of our knowledge, severe obstructive respiratory failure following inhalation of a gluten-free flour mixture containing hydrocolloids appears to be rarely reported. In our patient, acute respiratory failure following inhalation of gluten-free flour mixture was likely attributable to a combination of mechanical airway obstruction related to the physical properties of the inhaled material and its irritant effect on the bronchial mucosa. Accidental powder aspiration in children is a rare but potentially life-threatening event, for which current evidence consists predominantly of case reports. Severe respiratory impairment has been reported even following exposure to powders not conventionally considered toxic, including starches and food-grade products. Conversely, although hydrocolloids used as thickeners have been extensively studied for oral administration in children with dysphagia or reflux, data about the pulmonary effects and safety of their aspiration remain very limited.

This case emphasizes the value of a detailed clinical history in diagnosing rare causes of acute respiratory failure and highlights that seemingly trivial environmental exposures may have unexpected and potentially life-threatening consequences. Although the diagnosis was reached retrospectively and after a thorough review of the clinical history, the patient received appropriate management through therapeutic bronchoscopy with bronchoalveolar lavage, which successfully removed the obstructing material, resolved the atelectasis, restored adequate gas exchange, and ultimately allowed complete recovery with spontaneous breathing on room air.

## Patient perspective

Given his young age, the child was unable to provide a meaningful account of his perspective regarding the treatment received.

## Data Availability

The original contributions presented in the study are included in the article/supplementary material, further inquiries can be directed to the corresponding author.
